# Correlation of serum cartilage oligometric matrix protein (COMP) and interleukin-16 (IL-16) levels with disease severity in primary knee osteoarthritis: A pilot study in a Malaysian population

**DOI:** 10.1371/journal.pone.0184802

**Published:** 2017-09-14

**Authors:** Esha Das Gupta, Wei Ren Ng, Shew Fung Wong, Abdul Kareem Bhurhanudeen, Swan Sim Yeap

**Affiliations:** 1 Internal Medicine Department, School of Medicine, International Medical University, Seremban, Malaysia; 2 Department of Medicine, Subang Jaya Medical Centre, Subang Jaya, Selangor, Malaysia; University of Umeå, SWEDEN

## Abstract

**Objective:**

The aim of this study was to investigate the correlations between serum cartilage oligomeric matrix protein (COMP), interleukin-16 (IL-16) and different grades of knee osteoarthritis (KOA) in Malaysian subjects.

**Methods:**

Ninety subjects were recruited comprising 30 with Kellgren-Lawrence (K-L) grade 2 KOA, 27 with K-L grade 3 KOA, 7 with grade 4 KOA, and 30 healthy controls. All subjects completed the Western Ontario and McMaster Universities Arthritis Index (WOMAC) questionnaire. Serum COMP and IL-16 levels were measured using ELISA and their values log transformed to ensure a normal distribution.

**Results:**

There was no significant differences in levels of log serum COMP and IL-16 between healthy controls and KOA patients. There were no significant differences in the log serum COMP and IL-16 levels within the different K-L grades in the KOA patients. In KOA patients, log serum IL-16 levels significantly correlated with the WOMAC score (*p* = 0.001) and its subscales, pain (*p* = 0.005), stiffness (*p* = 0.019) and physical function (*p*<0.0001). Serum IL-16 levels were significantly higher in Malaysian Indians compared to Malays and Chinese (*p* = 0.024).

**Conclusions:**

In this multi-ethnic Malaysian population, there was no difference in serum COMP and IL-16 levels between healthy controls and patients with KOA, nor was there any difference in serum COMP or IL-16 levels across the various K-L grades of KOA. However, there were significant inter-racial differences in serum IL-16 levels.

## Introduction

Osteoarthritis (OA) is a “progressive disease of synovial joints that represents failed repair of joint damage that results from stresses that may be initiated by an abnormality in any of the synovial joint tissues, including articular cartilage, subchondral bone, ……… or synovium, which ultimately results in the breakdown of cartilage and bone” [1]. It is the commonest form of arthritis, affecting approximately 9.6% of men and 18% of women aged ≥ 60 years worldwide [2]. Due to the worldwide ageing population, OA is expected to be the fourth leading cause of disability by 2020 which would make it a significant health burden [2].Therefore, it would be useful if those at increased risk of OA could be identified early and preventive measures taken to reduce disease morbidity.

Although cartilage loss in OA is attributed to a mechanical failure of repair [3], recent studies have found synovial inflammation occurring in the OA joint [4]. This inflammation is thought to be responsible for the pain and swelling felt by some OA patients and is also critical in driving the pathological changes [4] such as negatively affecting the balance of cartilage matrix degradation and repair [3].

For many years, OA assessment has relied on using plain radiograph scoring systems of which the Kellgren-Lawrence (K-L) grading system is the most well-known [5]. However, any change to the x-ray images take a long time to become apparent, and x-ray changes do not always correlate with the symptoms which change more quickly. Assessment of cartilage in OA using MRI is now well-established [6], but access to such evaluations is limited by availability and cost. The WOMAC (Western Ontario and McMaster Universities Arthritis Index) questionnaire [7] assesses patient symptoms and is more responsive to changes in patients’ well-being but does provide information on what is happening at the joint/tissue level. Thus, there have been the development of biochemical markers or biomarkers, which can be easily measured in blood samples. These biomarkers have been used to try to make an early diagnosis of OA, for prevention of progression, or to measure tissue damage happening in the joint, to assess progression. Studies of biomarkers in osteoarthritis initially focused on cartilage degradation products, but now with the demonstration of inflammation within OA joints, inflammatory cytokines have also been studied.

Serum cartilage oligomeric matrix protein (COMP) is a non-collagen biomarker for cartilage degradation. Based on the results of a systematic review and meta-analysis, serum COMP was found to be consistently elevated in patients with radiographic knee OA (KOA) compared to healthy controls. In addition, higher levels of serum COMP are associated with a trend toward greater radiographic OA severity when compared to controls [8].

Pro-inflammatory cytokines have been implicated in the pathophysiology of OA, including IL-1β, TNF, IL-6, IL-15, IL-17, IL-18, IL-21 and IL-8 [9]. IL-16 was not previously considered to be involved in OA until in 2015, 2 papers from China showed that certain polymorphisms in the IL-16 gene influenced the susceptibility to KOA [10,11]. IL-16 is a pro-inflammatory cytokine whose functions include chemoattraction and modulation of T cell activation [12]. By binding with CD4 molecule, it activates CD4+ T cells, monocytes, macrophages, and eosinophils [13] and has been shown to be secreted in low levels from OA synovial fibroblasts [14]. Thus, IL-16 could be a potential mediator in the inflammatory process present in OA. Since IL-16 gene polymorphisms, and potentially, IL-16 production, could be under genetic control, it would be useful to study it in different populations of patients with KOA.

There have been no previous studies of serum biomarkers in Malaysian patients with KOA. We therefore set out to study serum COMP, a well-established marker in OA, in our multi-ethnic population to assess if its level was different in KOA patients compared to healthy controls, and if there was any difference in levels with KOA disease severity. In addition, we performed an exploratory study measuring serum IL-16, a novel pro-inflammatory mediator, in the same population, with a view to trying to replicate the other 2 studies as well as getting information on IL-16 levels in a multi-ethnic population.

## Materials and methods

### Subjects

Between November 2015 and March 2016, patients with primary KOA, over the age of 50 years, from the Orthopaedic outpatient clinics at Hospital Tuanku Ja’afar, Seremban, and Mawar Hospital, Seremban, were invited to participate in this study. Inclusion criteria were that patients had KOA as per the American College of Rheumatology clinical classification criteria using history and physical examination [15] and had an evaluable knee x-ray taken within the past 6 months. Patients with any other types of arthritis or secondary KOA were excluded. The x-rays were graded using the Kellgren-Lawrence (K-L) system from grade 0 (none), 1 (doubtful), 2 (minimal), 3 (moderate) to 4 (severe) [5] by a Consultant Radiologist and their Consultant Orthopaedic Surgeon as part of their routine clinical care. To ensure that the patients had definite radiological OA, patients with K-L grade 1 x-rays were excluded. Thirty normal controls over the age of 50 years, without clinical signs of KOA were recruited from the patients’ relatives attending Orthopaedic outpatient clinics from the same hospitals.

All subjects completed the WOMAC (Western Ontario and McMaster Universities Arthritis Index) questionnaire; Version LK 3.1 English for Australia Index with the Likert scale was used. The WOMAC Index is a validated, self-administered questionnaire that is widely used in the evaluation of subjects with hip and knee osteoarthritis. It assesses pain, stiffness and physical function through a set of 24 questions [7]. The higher the score, the worse the symptoms/function. The results range for the WOMAC questionnaire using the Likert scale was 0–96. For the WOMAC subscales, the range for pain was 0–20, stiffness 0–8 and physical function 0–68).

### Sample collection

2.5 mls of blood was withdrawn from each subject into a plain tube and allowed to clot at 4°C. The blood was then centrifuged for 15 minutes at 1,000 g, the supernatant serum was removed, aliquoted and stored at -20°C until use.

### Cytokine measurements

Serum COMP was measured using the Human COMP Quantikine ELISA Kit (R&D Systems), a solid phase sandwich ELISA immunoassay. The assay range was 0.2–10 ng/mL. The intra-assay precision co-efficient of variation (CV) was 2.1–3.8% and the inter-assay precision CV was 4.3–4.8%.Serum IL-16 was measured using the Human IL-16 Quantikine ELISA Kit (R&D Systems), a solid phase sandwich ELISA immunoassay. The assay range was 31.2–2,000 pg/mL. The intra-assay precision CV was 4.1–5.1% and inter-assay precision CV was 11.5–12%. Both assays were performed according to the manufacturer’s instructions.

### Statistical analysis

The sample size was calculated using an online sample size calculator (http://www.openepi.com/) with confidence interval set at 95% and power at 80%. The results obtained were 28 in each group for serum COMP levels, and 16 in each group for serum IL-16 levels. For ease of recruitment, the numbers in each group was rounded up to 30 and recruitment stopped for each group stopped once this number was reached.

Statistical analysis was done using IBM SPSS Statistics version 24 (IBM, Armonk, NY, USA). The variables were analysed for skewness and as suggested by Bulmer [16], the data was regarded as symmetrically distributed when the skewness values lay between -0.5 to +0.5. Age and BMI had skewness values of > +0.5, so non-parametric tests (Mann-Whitney and Kruskal-Wallis) were used to analyse their correlations with the other variables. Serum COMP and serum IL-16 also had skewness values of > +0.5. We therefore log transformed the data. With the log transformed data, the skewness value for log COMP was 0.010 and 0.054 for log IL-16 in keeping with a symmetrical distribution. The skewness value for the WOMAC score was 0.5. Thus parametric tests (one-way ANOVA and Pearson’s correlation) were used to assess the differences between log COMP, log IL-16 and WOMAC in the control and KOA group as a whole, and between the different K-L grades in the OA patients. Pearson’s correlation were used to analyse the correlations between WOMAC score, log COMP and log IL-16 levels. The Pearson Chi-Square test was used to compare the categorical variables. Statistical significance was assumed at two-sided *p* values at <0.05 level.

All subjects gave signed informed consent prior to inclusion in the study. This study was approved by the Joint Committee of the Research and Ethics Committee of the International Medical University and was conducted in accordance in accordance with the ethical standards laid down in the 2013 Declaration of Helsinki.

## Results

Ninety patients were recruited comprising 30 with K-L grade 2, 23 with K-L grade 3 and 7 with K-L grade 4 KOA, and 30 healthy controls. In the healthy control group there were 15 female and 15 male subjects. In the KOA group, there were 46 females and 14 males. There was significantly more females in the KOA group compared to controls (Pearson Chi-Square *p* = 0.011). There was no difference in the proportion of the different races (Chinese, Malay and Indian) between the 2 groups (Pearson Chi-Square *p* = 0.125). The median age was significantly lower in the control group compared to those with KOA, 57.50 vs 62.50 years (*p* = 0.001) with an age range of 40 years. There was no significant differences between the 2 groups with regards to their body mass index (BMI), log serum COMP and log serum IL-16 levels. The WOMAC and its subscales scores were significantly higher in the KOA patients compared to healthy controls (*p* < 0.0001). The comparisons between the healthy controls and KOA patients as a group are shown in Table 1. Males and females had similar log serum COMP and log serum IL-16 levels (*p* = 0.097, *p* = 0.399 respectively).

**Table 1:** Comparison between patients with knee osteoarthritis and healthy controls.

	KOA Patients	Healthy Controls	*p* value
	(n = 60)	(n = 30)	
Age (years)*	62.50 (12)	57.50 (9)	**0.001**^a^
Female (%)	46 (76.7%)	15 (50%)	**0.011**^a^
Race: (%)			**0.125^b^**
Chinese	17 (28.3%)	14 (46.7%)	
Malay	17 (28.3%)	9 (30.0%)	
Indian	26 (43.4%)	7 (23.3%)	
BMI (kg/m^2^)*	26.72 (5.35)	25.01 (6.31)	0.394^a^
WOMAC (range 0–96)**	45.25 ± 25.38	3.03 ± 5.57	**< 0.0001**
WOMAC pain (range 0–20)**	9.73±5.59	0.73±1.29	**< 0.0001**
WOMAC stiffness (range 0–8)**	3.00 ± 2.55	0.20 ± 0.61	**< 0.0001**
WOMAC physical function (range 0–68)**	32.52 ± 18.62	2.10 ± 4.53	**< 0.0001**
Log serum COMP**	0.258 ± 0.18	0.239 ± 0.10	0.581
Anti-log COMP mean (ng/mL)	1.81	1.73	N/A
Log serum IL-16**	2.167 ± 0.17	2.166 ± 0.19	0.981
Anti-log IL-16 mean (pg/mL)	146.89	146.55	N/A

KOA = knee osteoarthritis

BMI = body mass index

WOMAC = Western Ontario and McMaster Universities Arthritis Index

COMP = cartilage oligomeric matrix protein

IL-16 = interleukin-16

N/A = not applicable

*Values are given as median (interquartile range)

**Values are given as mean ± 1 standard deviation

*p* values are from One-way ANOVA test unless otherwise stated

a = Mann-Whitney U *p* value

b = Pearson Chi-Square *p* value

Bold *p*< 0.05 (statistically significant)

There was a significant positive correlation between WOMAC and BMI (Spearman’s r = 0.213, *p* = 0.044). In the WOMAC subscales, there were significant correlations between the pain (Spearman’s *p* = 0.023) and physical function (Spearman’s *p* = 0.048) but not the stiffness (Spearman’s *p* = 0.364) subscales. Log serum COMP significantly correlated with age (Spearman’s *p* = 0.005). There was no correlation between log serum COMP and IL-16 (Pearson correlation *p* = 0.111), even after correcting for age (Pearson correlation *p* = 0.122).

The KOA patients were then divided into their K-L grade for analysis and the results are shown in Table 2. The BMI was significantly higher in those with K-L grade 4 with a median of 29.91 kg/m^2^, compared to medians of 26.44 and 25.71 kg/m^2^ in K-L grade 2 and 3 respectively (*p* = 0.02). There was no significant differences in WOMAC and its subscales score, log serum COMP and log serum IL-16 levels between the 3 groups.

**Table 2:** Comparison between the kellgren-lawrence grades of knee osteoarthritis.

	K-L Grade 2	K-L Grade 3	K-L Grade 4	*p* value
	(n = 30)	(n = 23)	(n = 7)	
BMI (kg/m^2^)	26.44 (5.03)	25.71 (5.49)	29.91 (7.45)	**0.02**
WOMAC (Range 0–96)	41.93 ± 30.31	47.04 ± 18.79	53.57 ± 21.20	0.509
WOMAC pain (range 0–20)	9.07 ± 6.78	10.26 ± 3.92	6.28 ± 4.95	0.641
WOMAC stiffness (range 0–8)	2.47 ± 2.64	3.43 ± 2.27	3.86 ± 2.91	0.254
WOMAC physical function (range 0–68)	30.40 ± 22.16	33.35 ± 13.96	38.86 ± 15.77	0.544
Log serum COMP	0.273 ± 0.16	0.224± 0.20	0.307 ± 0.17	0.453
Anti-log COMP mean (ng/mL)	1.87	1.67	2.01	N/A
Log serum IL-16	2.155 ± 0.18	2.160 ± 0.12	2.240 ± 0.22	0.461
Anti-log IL-16 mean (pg/mL)	142.89	144.54	173.78	N/A

K-L = Kellgren-Lawrence

BMI = body mass index

WOMAC = Western Ontario and McMaster Universities Arthritis Index

COMP = cartilage oligomeric matrix protein

IL-16 = interleukin-16

N/A = not applicable

Values are given as mean ± 1 standard deviation apart from BMI (median [IQR])

*p* values are from One-way ANOVA test apart from Kruskal-Wallis test used for BMI

Bold *p*< 0.05 (statistically significant)

**Fig 1:**
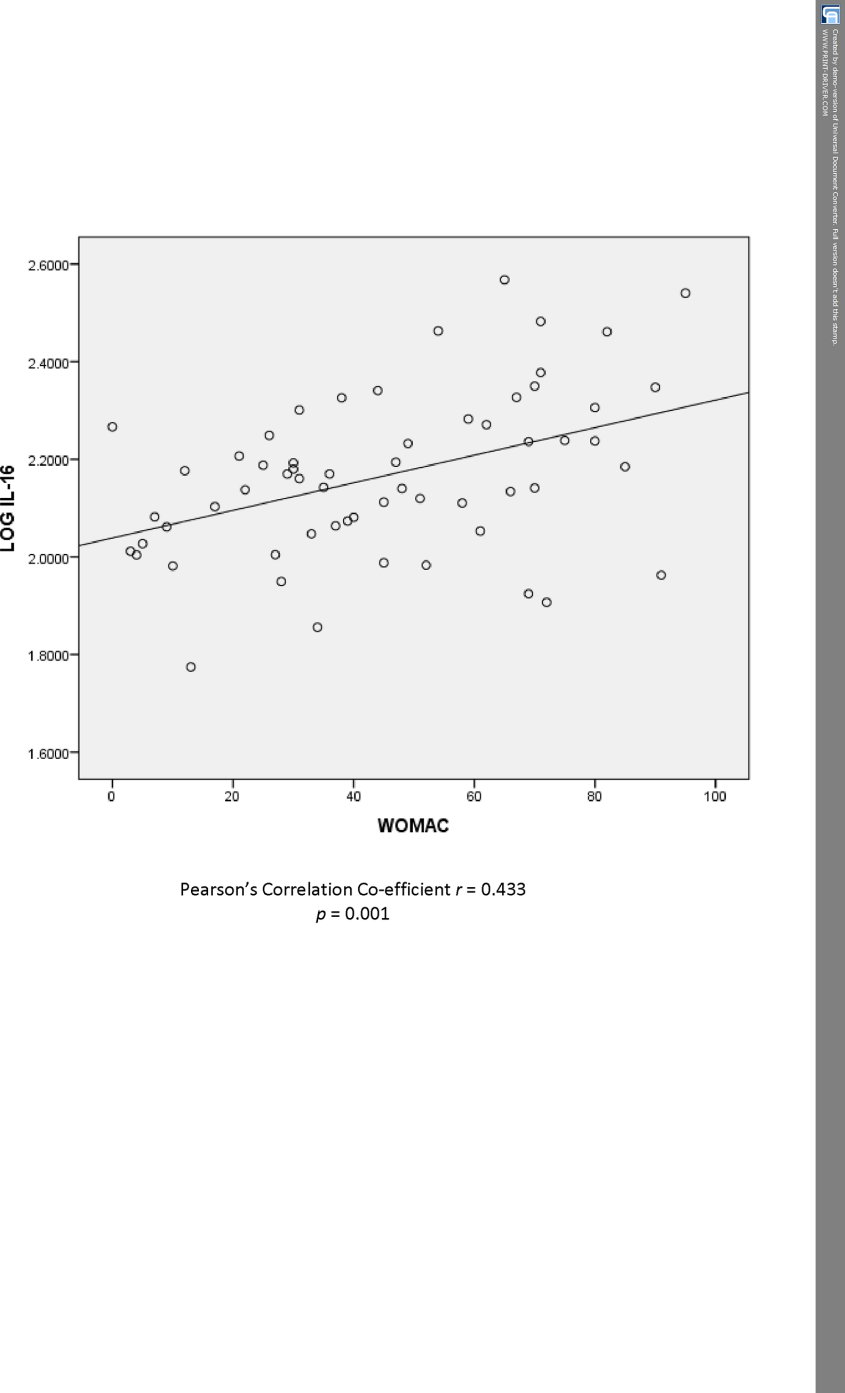
Correlation between IL-16 and WOMAC in patients with knee osteoarthritis.

Pearson’s correlation tests was performed to determine the correlation between WOMAC score and its subscales, log serum COMP and log serum IL-16 levels in patients with KOA. Log serum IL-16 was significantly correlated with WOMAC (Fig 1) (*p* = 0.001). Log serum IL-16 also significantly correlated with the WOMAC subscales–*p* = 0.005 for pain, *p* = 0.019 for stiffness and *p* < 0.0001 for physical function. There was no significant correlations between log serum COMP, WOMAC scores or its subscales (all *p* > 0.05). Log serum COMP significantly correlated with age (Spearman’s *p* = 0.001).

**Fig 2:**
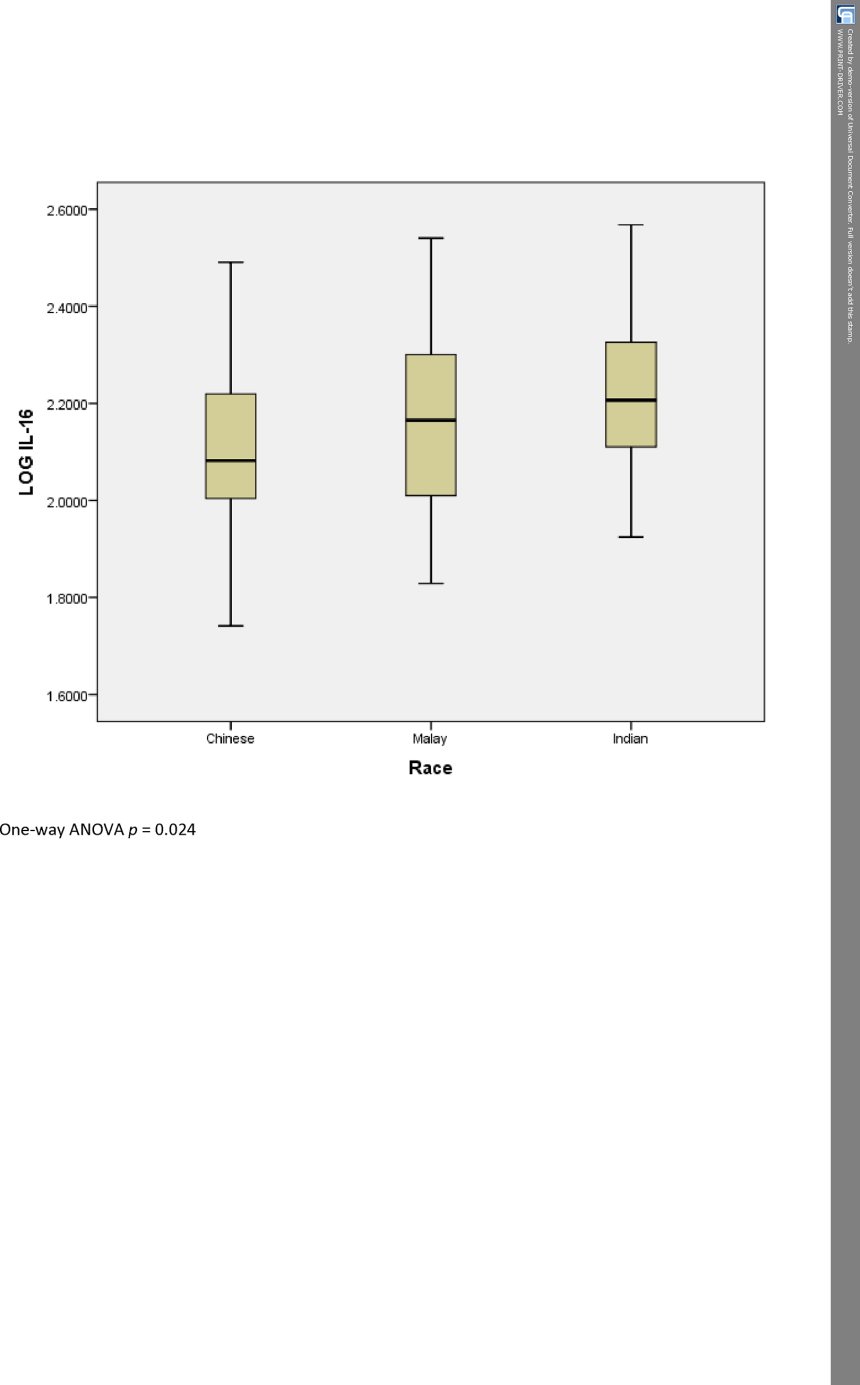
Boxplot of serum IL-16 levels between the different races.

Table 3 shows the characteristics of the 3 ethnic groups studied. Serum IL-16 levels, but not serum COMP levels, were significantly different between the various ethnic groups. The Indians had the highest levels of serum IL-16, followed by the Malays, followed by the Chinese who had the lowest levels (Fig 2). The Indians also had the highest BMI and WOMAC scores among the 3 races.

**Table 3:** Characteristics of the different races.

	Chinese	Malay	Indian	*p* value
Age (years)	62 (8)	57 (12)	61 (10)	0.167 ^a^
BMI (kg/m^2^)	24.44 (19.56)	27.51 (6.06)	27.70 (6.90)	**0.003** ^a^
WOMAC (Range 0–96)	17.19 ± 19.45	31.27 ± 30.49	44.24 ± 29.72	**0.001**
WOMAC pain (range 0–20)	3.58 ± 4.50	6.88 ± 6.11	9.58 ± 6.61	**< 0.0001**
WOMAC stiffness (range 0–8)	1.39 ± 1.91	1.96 ± 2.69	2.79 ± 2.68	0.76
WOMAC physical function (range 0–68)	12.23 ± 13.75	22.42 ± 22.33	31.88 ± 21.78	**0.001**
Log serum COMP	0.261 ± 0.13	0.269 ± 0.16	0.229 ± 0.17	0.574
Anti-log COMP mean (ng/mL)	1.82	1.85	1.69	N/A
Log serum IL-16	2.107 ± 0.16	2.163 ± 0.19	2.224 ± 0.15	**0.024**
Anti-log IL-16 mean (pg/mL)	127.94	145.55	167.49	N/A

BMI–body mass index

COMP = cartilage oligomeric matrix protein

IL-16 = interleukin-16

N/A = not applicable

Values are given as mean ± 1 standard deviation apart from age and BMI which are given as median (interquartile range)

*p* values are from One-way ANOVA test unless otherwise stated

a = Kruskall-Wallis *p* value

Bold p < 0.05 (statistically significant)

## Discussion

To our knowledge, this is the first study of serum biomarkers in KOA in a multi-ethnic Malaysian population. As cytokines are produced by various immune cells to modulate the cell-mediated and humoral response, they are more commonly studied in inflammatory conditions/inflammatory arthritides rather than in OA. The only other paper looking at cytokines in KOA published from Malaysia studied mRNA expression of IL-6 in KOA cartilage and found it no different from that in normal cartilage [17]. In other populations, the cytokines that have been studied in OA include IL-6 [18,19,20], IL-1α, IL-1β, TNFα, IL-18 [18,19], IL-2 [18,20], IL-10, IL-15 [19], IL-4 [20] and IL-5 [18]. Therefore, prior to the study by Luo and colleagues [11], IL-16 had not been studied in relationship to OA. In their study in Han Chinese, serum IL-16 levels were found to be 36.70 ± 6.72 pg/mL in healthy controls and 44.32 ± 8.78 pg/mL in KOA patients, which was significantly different (*p* = 0.001). As shown in Table 2, our study did show a trend towards a higher mean (anti-log serum) IL-16 level in those with K-L grade 4 which did not reach statistical significance. In addition, the levels of serum IL-16 in our population were lower, with mean (anti-log serum) levels of 146.55 pg/mL in the controls and 146.89 pg/mL in KOA patients, which were not significantly different. Even if we just looked at the Malaysian Chinese population, the mean (anti-log) serum IL-16 level was still higher at 127.94 pg/mL. One reason for the difference could be that different kits were used to measure the serum IL-16. In the study by Luo and colleagues [11], the lower limit of detection for serum IL-16 was 5 pg/mL whereas our lower limit of detection was 31.2 pg/mL, but both kits used the same unit of measurement. Nevertheless, it would suggest that there may be other influences on IL-16 levels apart from genetics, especially since Luo and colleagues [11] did not find any link between the IL-16 polymorphisms and IL-16 levels.

In a systematic review and meta-analysis, serum COMP was found to be elevated in patients with radiographic knee OA compared to healthy controls and higher levels are associated with more severe disease [8]. However, serum COMP has not been consistently found to have a predictive value for future development of OA. Kluzek and colleagues found in a British community-based cohort, the highest quartile of serum COMP was predictive of the development of radiographic and painful KOA [21]. In contrast, Kraus and colleagues did not find serum COMP to be useful as a predictor of worsening KOA [22]. Unlike the systematic review and meta-analysis, our study showed that serum COMP levels were similar between Malaysian KOA patients and controls, and similar in the different K-L grades of KOA severity. There have not been many studies in Asian populations. A study from Japan showed that serum COMP levels were higher in those with radiological OA compared to healthy controls [23]. A study from India did not show a correlation between serum COMP and radiological grading, similar to this study, although their patients with KOA had higher serum COMP levels compared to the controls [24]. Like this study, Verma and colleague [24] found that COMP was significantly associated with age, but unlike them we did not find a difference in levels between males and females. A study from China showed that more severe cartilage damage as determined at arthroscopy or arthroplasty was associated with higher serum COMP levels [25]. Our study cannot be directly compared to that of Jiao and colleagues [25] as we used plain x-rays as a surrogate measure of cartilage damage, rather than direct visualisation. However, as seen in Table 2, our study did find a trend towards a higher level of serum COMP in patients with K-L grade 4 but it did not reach statistical significance.

The WOMAC questionnaire assess pain, stiffness and physical function in patients with OA. Overall, the WOMAC score was significantly higher in KOA patients compared to healthy controls which was not unexpected. However, the mean (anti-log) levels of IL-16 were not different in the 2 groups. In contrast, there was a strong correlation between the WOMAC score and serum IL-16 and the WOMAC score and BMI when directly compared. While there are no previous studies evaluating the correlation between serum IL-16 and WOMAC, it can be hypothesised that the positive correlation found in our study between the two variables could suggest that inflammation as measured by serum IL-16 influences pain and physical function as assessed by WOMAC. There have been other studies that have shown a link between WOMAC scores and the pro-inflammatory cytokines, IL-6 [26], IL-15 [27] and TNFα [28,29]. Thus, it is not unexpected that IL-16, a pro-inflammatory cytokine has a positive correlation with the WOMAC score.

The positive correlation between WOMAC and BMI found in our study has been shown in previous studies. This is not unexpected as obesity is a well-known risk factor for OA [30]. A higher BMI was associated with worse KOA symptoms for all WOMAC and SF-36 subcategories [31] as well as radiographic damage [32].

One of the limitations to this study is that the sample size was small, but a sample size calculation was done so the numbers were adequately powered to show a difference. When comparing between the healthy controls and KOA patients, the numbers should have been adequately powered to show a difference as the calculations showed that only 28 patients were required in each group for the COMP and 16 patients for IL-16. As we had 30 controls and 60 patients, we should have been able to show a difference between the controls and KOA patients but we did not. However, the numbers of patients with K-L grade 4 KOA was small which may have affected the results when comparing the biomarker values with KOA disease severity.

We note that our control subjects had a positive WOMAC score. This may be because our control subjects are older, rather than younger. However, there have been 2 studies that have looked at the WOMAC score in a “normal” population. In the first study, the average score for physical function for both males and females was 1.78 [33]. In the other study, the average score for physical function for both males and females was 1.54, pain 1.41 and stiffness 2.01 [34]. This would be in keeping with our results that showed the average pain score of 0.73, average stiffness score of 0.20 and average physical function score of 2.10.

An interesting result of this study was to find that the serum IL-16 levels are different in the 3 major ethnic groups in Malaysia, and that the levels we have found are different from that found in Luo and colleagues’ study [11]. We feel that this warrants further study in a larger sample size and will be applying for funding to analyse the IL-16 gene polymorphisms and serum IL-16 levels in our multi-ethnic population.

## Conclusion

This is the first study looking at serum biomarkers in Malaysian patients with KOA. Two biomarkers were studied–serum COMP which has been well-studied in different KOA populations but not so commonly in Asian populations, and serum IL-16, a recently suggested OA biomarker of which there has been only 2 prior publications. We found that there was no difference in serum COMP and serum IL-16 levels between healthy controls and patients with KOA, and no significant difference in levels of both biomarkers across the various K-L grades of KOA. There was a significant correlation between WOMAC and serum IL-16 which may suggest that serum IL-16 levels can correlate with symptoms. Of interest is that serum IL-16 levels are significantly different between the 3 major ethnic groups in Malaysia, which warrants further investigation.

## Supporting information

S1 File Supplementary data file. (PDF)
