# Correction: Correlation of serum cartilage oligometric matrix protein (COMP) and interleukin-16 (IL-16) levels with disease severity in primary knee osteoarthritis: A pilot study in a Malaysian population

**DOI:** 10.1371/journal.pone.0190542

**Published:** 2017-12-27

**Authors:** Esha Das Gupta, Wei Ren Ng, Shew Fung Wong, Abdul Kareem Bhurhanudeen, Swan Sim Yeap

The word “oligomeric” is misspelled in the article title. The correct title is: Correlation of serum cartilage oligomeric matrix protein (COMP) and interleukin-16 (IL-16) levels with disease severity in primary knee osteoarthritis: A pilot study in a Malaysian population. The correct citation is: Das Gupta E, Ng WR, Wong SF, Bhurhanudeen AK, Yeap SS (2017) Correlation of serum cartilage oligomeric matrix protein (COMP) and interleukin-16 (IL-16) levels with disease severity in primary knee osteoarthritis: A pilot study in a Malaysian population. PLoS ONE 12(9): e0184802. https://doi.org/10.1371/journal.pone.0184802
