# Supplementary material for: Correlation of serum cartilage oligometric matrix protein (COMP) and interleukin-16 (IL-16) levels with disease severity in primary knee osteoarthritis: A pilot study in a Malaysian population
Source: PLoS One. 2017 Sep 14;12(9):e0184802. doi: 10.1371/journal.pone.0184802 (PMC5599033; doi:10.1371/journal.pone.0184802)
Supplement: S1 File — (PDF) [file pone.0184802.s001.pdf]

Grade II

Gender	Race	Age	OA Grade/Control	Weight	Height	BMI	WOMAC	PAIN	STIFFNESS	PHYSICAL FUNCTION	COMP	IL-16	LOG COMP	LOG IL-16
F	3	52	II	81	1.6	31.6	70	19	0	51	1.387	138.4292	0.142076461	2.141227834
M	2	71	II	79	1.65	29.0	25	5	1	19	2.544	154.1481	0.405517107	2.187938099
F	3	55	II	60	1.65	22.0	85	18	5	62	1.316	153.0197	0.119255889	2.18474727
F	2	54	II	67	1.57	27.2	51	10	4	37	1.5	131.7466	0.176091259	2.11973946
M	1	63	II	69	1.66	25.0	17	1	0	16	1.683	126.7571	0.226084116	2.102972156
F	1	60	II	59	1.63	22.2	29	4	3	22	1.502	147.9524	0.176669933	2.170122022
F	3	62	II	74	1.57	30.0	54	10	5	39	1.435	290.3912	0.156851901	2.462983404
F	2	57	II	72	1.58	28.8	34	9	0	25	2.359	71.78514	0.372727941	1.856034545
F	2	57	II	95	1.5	42.2	71	13	4	54	2.047	303.6637	0.311117843	2.482392934
M	3	57	II	76	1.65	27.9	21	8	3	10	1.578	160.9359	0.198106999	2.206653035
F	3	60	II	60	1.6	23.4	71	14	0	57	2.342	238.5287	0.369586891	2.377540648
M	3	67	II	67	1.57	27.2	39	6	2	31	1.496	118.4856	0.174931594	2.073665679
M	3	71	II	70	1.73	23.4	59	15	4	40	3.804	191.6572	0.580240508	2.282525204
F	3	69	II	50	1.57	20.3	9	2	0	7	0.966	115.194	-0.01502287	2.061429717
F	1	65	II	55	1.55	22.9	4	1	0	3	2.524	100.8967	0.402089351	2.003876894
F	1	50	II	75	1.62	28.6	13	4	1	8	1.395	59.49991	0.144574208	1.774516287
M	3	56	II	100	1.7	34.6	80	19	0	61	2.39	172.7328	0.378397901	2.237374847
F	2	50	II	70	1.6	27.3	67	14	7	46	1.028	212.3299	0.011993115	2.327011133
F	1	70	II	63	1.55	26.2	28	7	0	21	2.545	89.02806	0.405687787	1.949526902
F	1	75	II	54	1.5	24.0	0	0	0	0	3.212	184.7354	0.506775537	2.266550102
F	2	50	II	63	1.65	23.1	49	11	7	31	2.182	170.8184	0.338854746	2.232534749
F	1	56	II	70	1.7	24.2	5	1	1	3	2.227	106.4131	0.347720217	2.026995247
M	2	51	II	70	1.56	28.8	10	1	0	9	1.74	95.86094	0.240549248	1.981641694
F	1	60	II	55	1.5	24.4	33	8	3	22	2.057	111.49	0.313234292	2.047236005
F	1	66	II	60	1.57	24.3	7	0	1	6	1.652	120.7676	0.218010043	2.081950573
F	2	54	II	104	1.55	43.3	95	20	8	67	3.96	347.053	0.597695186	2.540395824
F	2	65	II	62	1.57	25.2	3	2	0	1	1.816	102.7329	0.259115844	2.011709527
F	3	65	II	60	1.5	26.7	90	19	8	63	2.482	222.5709	0.394801777	2.347468438
F	3	53	II	68	1.52	29.4	48	11	3	34	0.855	138.0794	-0.06803389	2.140128966
F	2	67	II	60	1.53	25.6	91	20	4	67	2.02	91.75637	0.305351369	1.962636226

Grade III/IV

Gender	Race	Age	OA Grade/Control	Weight	Height	BMI	WOMAC	PAIN	STIFFNESS	PHYSICAL FUNCTION	COMP	IL-16	LOG COMP	LOG IL-16
M	3	57	III	73	1.8	22.5	45	10	2	33	2.464	129.5266	0.391640703	2.11235903
F	2	62	III	65	1.6	25.4	35	9	1	25	0.617	138.7949	-0.20971484	2.142373492
F	3	90	IV	68	1.52	29.4	65	13	4	48	4.324	369.643	0.635885685	2.567782545
F	3	61	III	70	1.52	30.3	40	12	4	24	0.983	120.5288	-0.00744648	2.081090657

M	2	64	III	80	1.58	32.0	26	8	0	18	2.073	177.4587	0.316599302	2.249097395
F	2	52	III	82	1.75	26.8	66	14	3	49	1.179	136.198	0.071513805	2.134170646
F	1	60	III	64	1.6	25.0	45	9	4	32	1.933	97.26148	0.286231854	1.987940852
F	3	68	III	76	1.61	29.3	37	9	3	25	2.166	115.7413	0.335658452	2.063488275
F	1	80	IV	67	1.52	29.0	27	6	4	17	1.717	101.0391	0.234770295	2.004489469
F	3	63	III	78	1.5	34.7	58	11	6	41	1.505	128.9722	0.1775365	2.11049618
F	1	70	III	63	1.6	24.6	30	5	4	21	1.914	151.3287	0.281941933	2.179921185
F	1	66	III	60	1.52	26.0	36	7	3	26	1.382	147.9524	0.140508043	2.170122022
F	1	76	III	65	1.55	27.1	52	11	2	39	4.258	96.18486	0.629205657	1.983106724
F	3	71	III	59	1.65	21.7	61	11	5	45	1.843	113.0035	0.265525335	2.053091986
F	2	68	III	52	1.5	23.1	44	6	3	35	1.749	219.141	0.242789809	2.340723574
M	2	75	IV	65	1.57	26.4	31	7	0	24	2.375	199.9261	0.375663614	2.300869578
M	2	64	III	83	1.87	23.7	22	6	0	16	3.659	137.3131	0.563362409	2.137712118
F	3	56	IV	91	1.58	36.5	80	20	7	53	1.72	202.2456	0.235528447	2.30587904
M	2	68	IV	98	1.81	29.9	72	13	7	52	2.319	80.71732	0.365300749	1.906966747
F	3	63	III	64	1.52	27.7	12	3	0	9	0.871	150.2024	-0.05998184	2.176676818
F	3	61	IV	85	1.57	34.5	62	10	0	52	1.637	186.6431	0.214048679	2.271012028
M	3	53	III	67	1.52	29.0	69	14	5	50	1.893	172.3135	0.277150614	2.236319325
F	3	62	III	85	1.56	34.9	70	17	6	47	1.511	223.8256	0.179264464	2.349909767
F	1	65	III	64	1.65	23.5	47	17	2	28	1.415	156.4074	0.15075644	2.194257258
F	1	76	III	56	1.56	23.0	30	8	3	19	3.001	155.8422	0.477265995	2.192685196
M	3	76	III	67	1.7	23.2	82	14	5	63	2.391	289.2505	0.378579576	2.4612741
F	3	62	IV	92	1.54	38.8	38	7	5	26	1.232	211.8432	0.090610708	2.326014501
F	3	64	III	60	1.57	24.3	31	6	2	23	0.991	144.6765	-0.00392635	2.160397955
F	3	53	III	74	1.65	27.2	69	16	8	45	1.126	84.04355	0.051538391	1.924504372
F	1	58	III	70	1.65	25.7	75	13	8	54	1.629	173.2117	0.211921084	2.238577154

Controls

Gender	Race	Age	OA Grade/Control	Weight	Height	BMI	WOMAC	PAIN	STIFFNESS	PHYSICAL FUNCTION	COMP	IL-16	LOG COMP	LOG IL-16
M	1	52	Control	61	1.63	23.0	4	0	1	3	1.193	109.7271	0.076640444	2.040313917
M	3	54	Control	76	1.53	32.5	14	2	0	12	2.65	108.0926	0.423245874	2.033795946
M	3	51	Control	76	1.62	29.0	0	0	0	0	2.616	266.4181	0.41763774	2.425563661
M	1	71	Control	62	1.65	22.8	0	0	0	0	1.52	102.2736	0.181843588	2.009763523
F	1	62	Control	61	1.5	27.1	9	2	0	7	1.506	55.11499	0.177824972	1.741269772
F	1	63	Control	63	1.57	25.6	18	3	0	15	1.893	113.3408	0.277150614	2.054386223
M	2	53	Control	80	1.65	29.4	0	0	0	0	1.71	67.45706	0.23299611	1.829027435
F	3	61	Control	56	1.49	25.2	0	0	0	0	2.307	156.9914	0.363047595	2.19587575
M	2	55	Control	70	1.68	24.8	0	0	0	0	2.923	182.8107	0.465828815	2.262001582
M	2	57	Control	80	1.7	27.7	6	2	1	3	1.685	206.9811	0.226599905	2.31593067

F	2	62	Control	98	1.65	36.0	0	0	0	0	1.607	108.7185	0.206015877	2.036303346
M	3	62	Control	75	1.72	25.4	0	0	0	0	1.396	112.8779	0.144885418	2.0526088
M	1	52	Control	86	1.53	36.7	0	0	0	0	1.225	117.9782	0.088136089	2.07180168
M	2	58	Control	68	1.7	23.5	1	0	1	0	1.549	89.02806	0.190051418	1.949526902
M	2	50	Control	58	1.54	24.5	6	4	0	2	1.605	102.2736	0.205475037	2.009763523
F	2	56	Control	83	1.56	34.1	6	4	0	2	2.127	262.4132	0.32776749	2.418985644
F	1	63	Control	40	1.45	19.0	21	3	3	15	1.93	178.486	0.285557309	2.251604226
F	3	53	Control	55	1.5	24.4	1	0	0	1	1.752	287.0919	0.243534102	2.458020923
F	1	58	Control	57	1.52	24.7	0	0	0	0	1.27	309.4691	0.103803721	2.490617339
M	3	61	Control	73	1.74	24.1	0	0	0	0	1.743	180.4825	0.241297387	2.256435013
M	1	59	Control	73	1.6	28.5	1	0	0	1	2.219	177.8272	0.346157302	2.249998168
F	2	50	Control	75	1.56	30.8	0	0	0	0	1.673	167.7568	0.223495941	2.224680135
F	1	57	Control	43	1.55	17.9	0	0	0	0	2.208	97.77821	0.343999069	1.99024206
F	1	62	Control	50	1.59	19.8	0	0	0	0	1.908	173.0531	0.28057837	2.238179329
F	2	59	Control	90	1.63	33.9	2	1	0	1	1.483	192.7234	0.171141151	2.284934341
M	1	51	Control	63	1.65	23.1	0	0	0	0	1.451	176.235	0.161667412	2.246092276
F	1	57	Control	90	1.55	37.5	0	0	0	0	1.466	265.3503	0.16613397	2.423819663
F	3	51	Control	57	1.54	24.0	0	0	0	0	1.376	184.2034	0.138618434	2.265297569
F	1	58	Control	70	1.7	24.2	0	0	0	0	1.934	158.7738	0.28645647	2.200778954
M	1	61	Control	65	1.68	23.0	2	1	0	1	1.475	88.54926	0.16879202	1.947184925

Race key: 1 = Chinese
2 = Malay
3 = Indian
